# An occupational exposure limit for welding fumes is urgently needed

**DOI:** 10.5271/sjweh.4002

**Published:** 2021-12-30

**Authors:** Bengt Sjögren, Karin Broberg, Håkan Tinnerberg, Maria Albin, Per Gustavsson, Gunnar Johanson

**Affiliations:** Integrative Toxicology, Institute of Environmental Medicine, Karolinska Institutet, Stockholm, Sweden; Institute of Environmental Medicine, Karolinska Institutet and Division of Occupational and Environmental Medicine, Lund University Lund, Sweden [Email: karin.broberg@ki.se]; Institute of Medicine, Sahlgrenska Academy, School of Public Health, University of Gothenburg,and Community Medicine, Gothenburg, Sweden [Email: hakan.tinnerberg@amm.gu.se]; Unit of Occupational Medicine, Institute of Environmental Medicine, Karolinska Institutet, Stockholm, Sweden [Email: maria.albin@ki.se]; Institute of Environmental Medicine, Karolinska Institutet and Centre for Occupational and Environmental Medicine Stockholm, Sweden [Email: per.gustavsson@ki.se]; Integrative Toxicology, Institute of Environmental Medicine, Karolinska Institutet, Stockholm, Sweden [Email: gunnar.johanson@ki.se]

Approximately 11 million people work as welders worldwide and an additional 110 million are exposed to welding fumes at work (1). Several countries have an occupational exposure limit (OEL) for welding fumes of 5 mg/m^3^ (1, 2) and similar OEL for respirable dust (2). Given the accumulating evidence on serious health effects from welding fumes <5 mg/m^3^, adequate worker protection including a more stringent health based OEL is an urgent issue. We therefore welcome that the European Commission has assigned the European Chemical Agency (ECHA) to propose an OEL for welding fumes at the EU level, pursuant to the Carcinogens and Mutagens Directive (CAD). It should be noted that welding fumes – besides having a very complex and variable composition – are process generated and do not fall under the Registration, Evaluation, Authorisation and Restriction of Chemicals (REACH) regulation. In the following, we present some of the key issues when setting an OEL for welding fumes.

## Brief summary of health effects

The International Agency for Research on Cancer has classifed welding fumes as carcinogenic to humans (1). The evaluation supports chronic inflammation and immunosuppression rather than genotoxicity as a mechanism for welding-induced lung cancer. Meta-analyses have showed increased risks for lung cancer already after 3–20 years of exposure (3, 4).

Several epidemiological studies have shown increased risks for ischemic heart disease among welders (5), and a meta-analysis demonstrated increased risks of ischemic heart disease [risk ratio (RR) 1.09, 95% confidence interval (CI) 1.00–1.19, based on ten populations] as well as acute myocardial infarction (RR 1.69, 95% CI 1.18–2.42, based on three populations) (6). Ibfelt et al’s study (7) showed an increased risk at 10–50 mg/m^3^-years (the lowest exposure category, levels are given as respirable fraction unless otherwise stated). This corresponds to 0.25–1.25 mg/m^3^ during 40 years of welding (5). Welders with a median respirable dust exposure <1 mg/m^3^ (5–95 percentile ranges 0.2–4.2 and 0.1–1.9 at two time points) developed increased systolic and diastolic blood pressure (8).

Two recent studies provide data on chronic obstructive pulmonary disease (COPD) in relation to welding fumes. A significantly increased prevalence of COPD was seen among Korean welders in both the median and high exposure tertile. The exposure in the median tertile was 3.4–11.7 mg/m^3^-years, corresponding to 0.1–0.3 mg/m^3^ during 40 years (9). In a population-based cohort in Sweden, exposure to welding fumes was associated with an increased incidence of COPD at a mean exposure to inhalable dust of 0.8 mg/m^3^ but not at a mean exposure of 0.08 mg/m^3^ (10).

Mild steel contains small amounts of manganese [typically <1.6%, (1)], a known neurotoxicant (11). Gliga et al (12) found a strong correlation between respirable manganese and respirable dust during mild steel welding. According to their calculations, the current EU OEL for respirable manganese of 0.05 mg/m^3^ corresponds to 0.8 mg/m^3^ welding fumes.

Welding fume exposure has been associated with asthma (13, 14), with stainless steel welding fumes as a specific risk factor. In Finland, the estimated incidence of occupational asthma among stainless steel welders was 1–2 among 1000 welders/year (15). Several epidemiological studies have shown an increased frequency of pneumonia among welders. Welding fumes have also been associated with invasive pneumococcal disease (16). Exposure estimates associated with asthma and pneumonia are lacking.

Regarding effects on reproduction, a cohort following all single births in Sweden 1994–2012 showed that pregnant women with exposure to welding fumes (0.1–3.2 mg/m^3^) was associated with increased risks of pre-term birth and giving birth to children with low birth weight (17).

## Conclusion

As illustrated herein, data on several types of negative health effects from welding fumes at low-to-moderate exposure levels are available, and there is an urgent need for a health-based OEL for welding fumes. This OEL should be based on a critical appraisal of all health effects of welding and take the various welding methods into account. Indeed, some countries have already introduced such OEL, eg, Denmark (0.5–1.7 mg/m^3^ depending on welding process and material (18) and The Netherlands (1 mg/m^3^ ( 2, 19).

A general OEL for welding fumes does not replace the need for specific OEL for components such as chromium, nickel, aluminium, lead and manganese, which may be present to a variable extent depending on welding technique and material. The combined use of a general OEL and specific OEL makes it easier to ensure safe levels for different types of welding. Moreover, setting an OEL is not enough. Additional measures include local exhaust ventilation and fresh-air respirators. Furthermore, the health risks mentioned above as well as the ventilation measures need to be clearly communicated, for example in safety data sheets added to packages of welding electrodes. Furthermore, welders over the age of 50 may be recommended to vaccinate against pneumococcal pneumonia (20).
